# Micronized Powder of Raspberry Pomace as a Source of Bioactive Compounds

**DOI:** 10.3390/molecules28124871

**Published:** 2023-06-20

**Authors:** Renata Różyło, Ryszard Amarowicz, Michał Adam Janiak, Marek Domin, Sławomir Gawłowski, Ryszard Kulig, Grzegorz Łysiak, Klaudia Rząd, Arkadiusz Matwijczuk

**Affiliations:** 1 Department of Food Engineering and Machines, University of Life Sciences in Lublin, Głęboka 28, 20-612 Lublin, Poland; slawomir.gawlowski@up.lublin.pl (S.G.); ryszard.kulig@up.lublin.pl (R.K.); grzegorz.lysiak@up.lublin.pl (G.Ł.); 2 Department of Chemical and Physical Properties of Food, Institute of Animal Reproduction and Food Research, Polish Academy of Sciences, Tuwima 10, 10-748 Olsztyn, Poland; r.amarowicz@pan.olsztyn.pl (R.A.); m.janiak@pan.olsztyn.pl (M.A.J.); 3 Department of Biological Bases of Food and Feed Technologies, University of Life Sciences in Lublin, 28 Głęboka Str., 20-612 Lublin, Poland; marek.domin@up.lublin.pl; 4 Department of Biophysics, Institute of Molecular Biophysics, Faculty of Environmental Biology, University of Life Sciences in Lublin, Akademicka 13, 20-950 Lublin, Poland; k.terlecka.98@wp.pl (K.R.); arkadiusz.matwijczuk@up.lublin.pl (A.M.); 5 ECOTECH-COMPLEX—Analytical and Programme Centre for Advanced Environmentally-Friendly Tech-Nologies, Maria Curie-Sklodowska University, Głęboka 39, 20-033 Lublin, Poland

**Keywords:** micronization, raspberry pomace, FTIR spectra, phenolic identification, antioxidant activity

## Abstract

Red raspberries, which contain a variety of nutrients and phytochemicals that are beneficial for human health, can be utilized as a raw material in the creation of several supplements. This research suggests micronized powder of raspberry pomace production. The molecular characteristics (FTIR), sugar, and biological potential (phenolic compounds and antioxidant activity) of micronized raspberry powders were investigated. FTIR spectroscopy results revealed spectral changes in the ranges with maxima at ~1720, 1635, and 1326, as well as intensity changes in practically the entire spectral range analyzed. The discrepancies clearly indicate that the micronization of the raspberry byproduct samples cleaved the intramolecular hydrogen bonds in the polysaccharides present in the samples, thus increasing the respective content of simple saccharides. In comparison to the control powders, more glucose and fructose were recovered from the micronized samples of the raspberry powders. The study’s micronized powders were found to contain nine different types of phenolic compounds, including rutin, elagic acid derivatives, cyanidin-3-sophoroside, cyanidin-3-(2-glucosylrutinoside), cyanidin-3-rutinoside, pelargonidin-3-rutinoside, and elagic acid derivatives. Significantly higher concentrations of ellagic acid and ellagic acid derivatives and rutin were found in the micronized samples than in the control sample. The antioxidant potential assessed by ABTS and FRAP significantly increased following the micronization procedure.

## 1. Introduction

Red raspberries (*Rubus idaeus* L.) contain a variety of nutrients and phytochemicals important to human health [1,2,3]. Raspberry consumption is effective in reducing the levels of oxidative and inflammatory stress that promote heart morphological changes in old age, thereby preventing or delaying heart disease [4]. These fruits are unique berries with a rich nutritional and bioactive composition. They are a source of several essential micronutrients and dietary fiber [5]. Raspberry fruit is rich in antioxidant compounds, especially polyphenols [6,7,8]. The polyphenols present in the fruit are mainly ellagitannins and anthocyanins. Due to the high content of anthocyanins, these fruits have a red color [5]. The antioxidant activity of raspberries is directly related to the total amount of phenolic compounds found in raspberries [9].

Many health benefits of raspberry fruit have been established. An increasing number of studies suggest that red raspberries may play a role in lowering the risk of metabolically related chronic diseases [5]. In a study on obese individuals with type 2 diabetes, the effects of earing raspberries daily were discovered to potentially lower postprandial hyperglycemia and inflammation in diabetic adults, as well as to have anti-inflammatory properties [10]. A diet high in raspberries has been demonstrated to improve immunological function and phospholipid metabolism in obese patients in trials [11]. Other clinical trials have demonstrated that including fresh raspberry extract in the diet of old rats lowers indicators of aging, improves psychomotor coordination and balance, and boosts muscle tone and endurance [12].

Red raspberries are commonly used for producing dietary supplements because of their health benefits. They can be purchased in the form of dried or liquid products [13]. Raspberry ketone (4-(4-hydroxyphenyl)-2-butanone) supplements have also gained popularity among customers due to their ability to burn fat and aid in weight loss. Raspberry ketone is found naturally in raspberry fruit (up to 4.3 mg/kg) [14] and is used as a flavoring substance [15]. Processing raspberries extends their shelf life and culinary uses, although the nutritional information among processed forms is limited [16,17]. Raspberries are often processed into juices. After producing the juices, the pomace is obtained, which is a recyclable by-product [18]. Fresh pomace is perishable; thus, drying it can significantly extend its shelf life and increase reusability. Raspberry powders obtained after the dehydration of whole fruit or pomace can be added as functional additives (e.g., natural dyes) to food preparations. In most studies, powders were obtained from drying whole fruits [19,20]; only a few studies concern the drying of raspberry pomace [21]. Previous findings suggest that powders obtained by spray-drying raspberry extracts could be used as natural colorants or antioxidants [22]. Drying studies of whole raspberries have shown that these techniques affect the physical properties, bioactive compounds, and antioxidant activity of the resulting powders differently [19]. Previous studies have shown that freeze-drying causes changes in the physical properties of raspberry pomace [21]. Only a few data describe the antioxidant properties of processed raspberry byproducts [22,23].

There is no information on micronized forms of raspberry byproducts. Whole raspberry fruit micronization tests were carried out only by fluidized bed jet milling with drying [24]. Micronization is a grinding process that reduces the particle size of material from microns down to the nanometer range. This technology has developed rapidly in food production in recent decades [25,26,27]. Previous research has proven that the micronization process can be applied to plant materials, resulting in increased functionality [28,29].

The process of micronization or fine grinding has been applied to other plant materials, and it has been demonstrated that techniques leading to particle size reduction also cause changes in powder properties such as viscosity and porosity, and such powders can be used for a variety of purposes [30]. 

Additionally, the use of infrared spectroscopy has provided insight into the structure/interactions or packing of molecules during this and other similar processes. This information can facilitate the identification of fast molecular markers indicative of such changes. Notably, there are still rather few publications on the topic available in the literature, although some authors are beginning to publish findings in this area. 

Micronization in our studies was carried out with a ball mill; such studies on raspberry pomace powders are missing in the available literature. The research hypothesis assumes that raspberry byproduct micronization with the use of a ball mill results in a significant reduction in particle size and may have a positive effect on the biochemical properties of the obtained powders. The obtained powders were analyzed using the technique of ATR/FTIR spectroscopy (attenuated total reflectance Fourier transform infrared spectroscopy) to identify any changes that could potentially occur at the molecular level during the process of micronization. The presented results will contribute to the identification of marker changes occurring at the molecular level during the analyzed process of powder micronization. Furthermore, the presented molecular analysis will facilitate the easy identification of such changes in subsequent studies pertaining to this and similar types of samples, even if the micronization process itself is modified.

## 2. Results

### 2.1. Particle Size and Color Results of Micronized Byproduct Raspberry Powders

**Figure 1:**
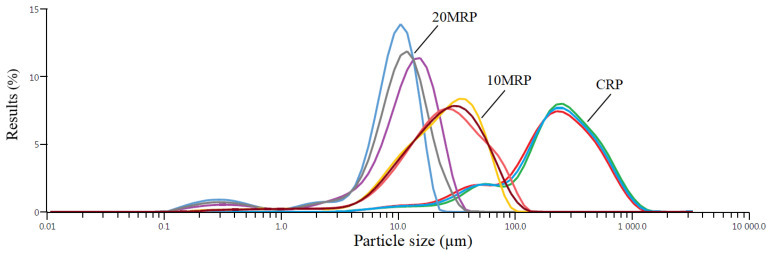
Particle size distribution for raspberry byproduct powders. CRP—control (without micronization) raspberry powder; 10 MRP—raspberry byproduct micronized for 10 min; 20 MRP—raspberry byproduct micronized for 20 min. Different line colors grouped with the same name mean single measurements. Different line colors grouped with the same name mean single measurements.

The investigation demonstrated that, as expected, the micronization process with the utilized ball mill induced considerable changes in the particle size distribution of the studied samples (Figure 1). The mean particle size (D[4;3]) was equal to 277 μm for the control sample (CRP). After 10 min (10 MRP) of micronization, this value dropped to 29.8 μm, and after 20 min of micronization (20 MRP) to 11 μm. More than 90% of the particles (d90) in the control sample (CRP) had dimensions below 578 μm. In the sample with 10 min of micronization, this parameter was 59.4 μm, and with 20 min of micronization, only 19.2 μm. The particle sizes for the 50% share (d50) were less than 225 μm for CRP, 25 μm for 10 MRP, and 10.5 μm for 20 MRP.

In other studies in which superfine grinding of apple pomace [31] or herbal plant [32] was used, similarly to our tests, a significant reduction in particle size was observed. In these tests, the control apple pomace had a d50 particle size below 326 μm, and fine grinding reduced the d50 particle size parameter below 51.5 μm. In our research, at 10 and 20 min of micronization (10 MRP; 20 MRP), we obtained much lower dimensions of d50, i.e., 25 and 10.5 μm. This was due to different characteristics of the raw material. Here, we micronized raspberry pomace after removing the seeds. For cryogrinding buckwheat hulls [33], the d50 was below 15.1 μm. Micronization of oat husks [34] helped to obtain d50 dimensions below 15.5 μm.

**Figure 2:**
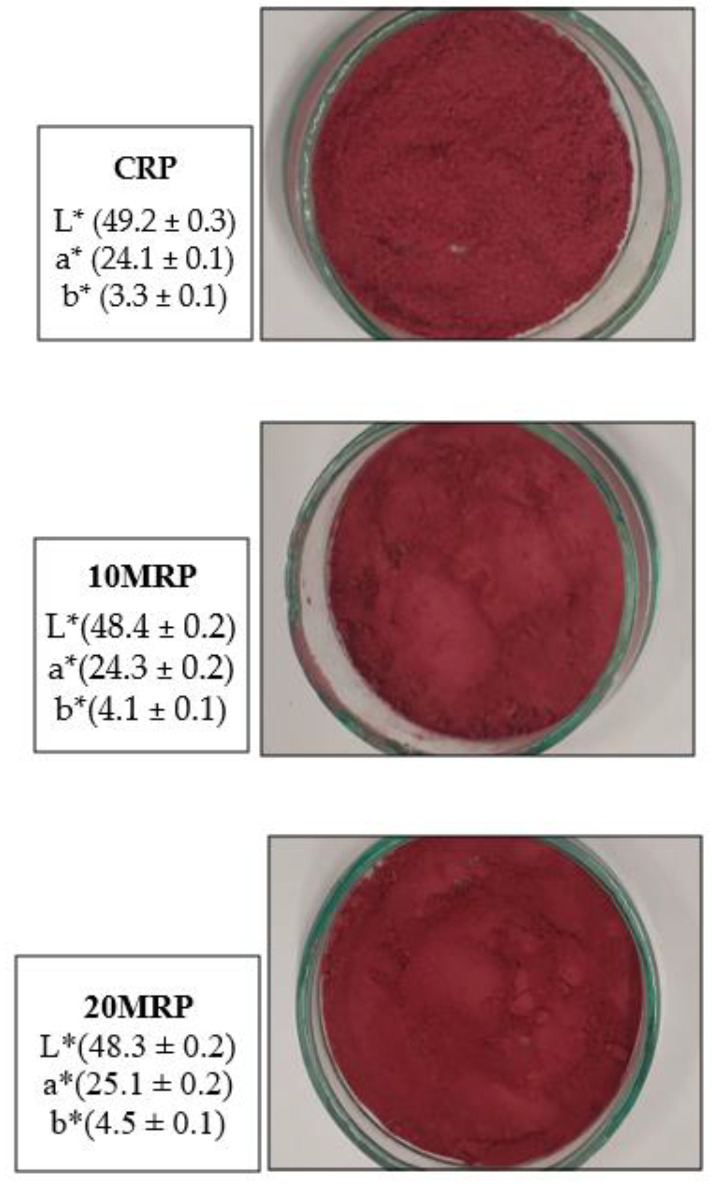
Appearance and color parameters of raspberry byproduct powders. CRP—control (without micronization) raspberry powder; 10 MRP—raspberry byproduct micronized for 10 min; 20 MRP—raspberry byproduct micronized for 20 min. L*—brightness color parameter; a*—redness color parameter; b*—blueness color parameter.

The color results revealed that the color of the raspberry powders after micronization differed marginally, but this difference was significant, particularly in the case of the L* and b* parameters. The control (CRP) sample had the lightest color (L* = 49.2), and the longest micronized sample (20 MRP) had the darkest color (L* = 48.3) (Figure 2). 

There were no significant differences in this parameter between 10 and 20 min of micronization. For the longest time (20 MRP), the share of red color a* was highest in the micronized samples, and the share of yellow color b* was highest in the micronized samples (20 MRP). Previous research [28] has shown that the micronization method affects the color of spinach stems and leaves. In comparison to the control sample and dry micronization, wet micronization produced a darker color.

### 2.2. FTIR Results of Micronized Byproduct Raspberry Powders

**Figure 3:**
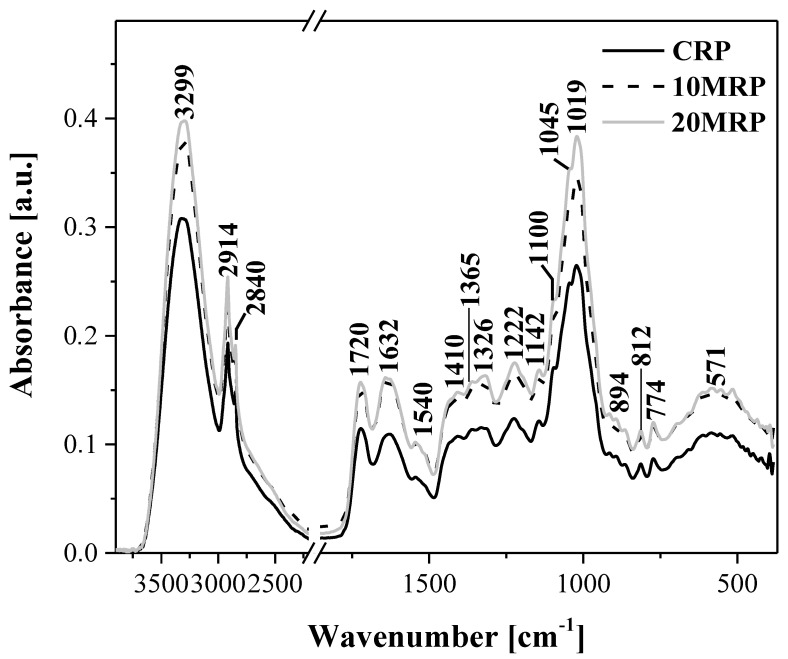
FTIR spectra for the analyzed raspberry samples: CRP—control (without micronization) raspberry powder; 10 MRP—raspberry byproduct micronized for 10 min; 20 MRP—raspberry byproduct micronized for 20 min. The measurements were performed within the spectral range from 450 to 3700 cm^−1^. The spectra were registered at room temperature (See Section 3).

At the next stage of the study, spectroscopic ATR-FTIR measurements were also performed (Figure 3), which assessed the impact of the sample treatment employed to facilitate more effective micronization. The results obtained from the spectral analysis in the infrared range might suggest that changes occur at the molecular level in the chemical structure of the raspberry samples in question.

Table 1 presents all the characteristic bands present in the obtained spectra and correlates them with specific vibrations of their respective functional groups, based on a detailed literature review [31,32,33,35,36,37,38,39,40,41] as well as on the careful structural analysis of the molecules present in our samples. 

The infrared spectra recorded for the analyzed raspberry byproduct contain clear bands that can be fairly accurately associated with identifiable vibrations of particular functional groups that are characteristic of ingredients present in products rich in polysaccharides and similar nutrients [31,32,33,38]. 

Starting from the highest wave numbers, we first observed characteristic, wide bands that are present in the range from approx. 2500 to 3600 cm^−1^. The maximum of these bands, located at ~3300 cm^−1^, is typical of the vibrations of asymmetric bonds induced by the stretching vibrations of the hydroxy -OH groups, which are characteristic of polysaccharide molecules that are predominant in the analyzed samples [38,41]. Moreover, the groups are involved in the formation of hydrogen bonds between smaller units of polysaccharide molecules. Notably, the band corresponding to the stretching vibrations of the -OH groups enhances the stretching vibrations of the C-H groups [36,41]. There are two very sharp bands present in this region, with wave numbers of 2914 and 2848 cm^−1^, that are particularly characteristic of asymmetric and symmetric stretching vibrations in the CH_2_ groups present in this type of food sample. The highest intensity of this particular band, similar to the band corresponding to the -OH vibrations, could be observed in the sample subjected to micronization for 20 min, with a lower value registered for the sample micronized for 10 min and the lowest for the control. 

Next, we proceed to the analysis of the essential group of vibrations known as the fingerprint region. The first two key bands corresponded to vibrations with the maxima at 1720 and 1632 cm^−1^, respectively. The former was associated with the characteristic stretching vibrations of the carbonyl group ν(C=O) [38] found in the molecules of simple sugars present in the samples. The latter band with the maximum at approx. 1630 cm^−1^ [37,38] corresponded to the deformation vibrations characteristic of water molecules, δ(-OH). During the micronization process, the relation between the two bands was noticeably altered. In the control, the respective ratio of the 1632/1720 bands was: 0.94, whereas in the sample subjected to 20 min., micronization increased to as much as 1.04. This pair of bands can therefore be treated as an excellent spectroscopic marker for the processing treatment in question. 

Furthermore, the bands observed in the region from 1550 to 900 cm^−1^ corresponded to the strong vibrations of the C-O, C-C, C-O-H, and C-O-C groups, various oligomolecules or polysaccharides [32,33]. Even though the spectra recorded in this region seemed similar in terms of shape, two significant changes ought to be noted. Firstly, there was a change in the shape and increase in the intensity of the band with the maximum at 1540 cm^−1^, characteristic of ν(C=C) vibrations. Secondly, we observed changes in terms of the intensity and, most importantly, the shape of the bands with the maximum at 1326 cm^−1^, characteristic of δ(C-H) vibrations and potentially enhanced by δ(O-H) vibrations in the molecules of poly- and oligosaccharides that were the primary ingredients of the analyzed samples. While other vibrations in this region retained their original shapes, their intensity was significantly increased with the growing duration of the micronization process. On addition, the intensity of the bands with the maximum at 1412 cm^−1^ was characteristic of δ(C-H) vibrations. Next, we observed an increase in intensity of the vibrations with the maxima at 1360, 1222, and 1144 cm^−1^. These are characteristic of the vibrations of, respectively, δ(-OH in plane), δ(CH2) groups, and δ(C-H) groups, as well as of the stretching vibrations in the C-O-C system found in oligo- and polysaccharides present in raspberries. We also observed increased intensity of the bands, with the main maximum at 1019 cm^−1^. The bands in this spectral region are primarily associated with C-O and C-O-C stretching vibrations in polysaccharide molecules. The highest intensity of these vibrations was observed in the samples subjected to 20 min processing. 

The last spectral “fingerprint” region below 930 cm^−1^ corresponds primarily to the crystalline regions and indicates conformational changes occurring in the analyzed material through possible changes to the β-1,4-glycoside bonds in polysaccharide molecules [38]. As we know, a fingerprint region is a spectral infrared range where each organic compound produces its unique absorption band. Such bands provide information as to the presence of various functional groups found in the given analyzed sample. As can be seen in our results, in the discussed case, the region below 930 cm^−1^ was characterized by a relatively low intensity of the bands with the maxima at ~891, 808, 774, or 580 cm^−1^. Apart from intensity variations, no particularly significant changes were observed here. Nonetheless, the changes in vibration intensity observed in this region, particularly in samples subjected to micronization, clearly indicate effects on the bonds between individual units in polysaccharide molecules [31,32,33]. 

To briefly recapitulate the already discussed results obtained from the spectral FTIR measurements, the observed discrepancies in terms of band intensity and, in some cases, slight shifts thereof indicate that the mechanical strength of finely ground material had a significant impact in the molecular properties of the analyzed samples. Firstly, the method of micronization employed for the raspberry byproduct samples resulted in cleaving the intramolecular hydrogen bonds in cellulose, hemicellulose, and polysaccharides predominantly present in the samples in question, likely leading to an increase in their content of amorphic cellulose and simple saccharides [31,32,33]. This was evidenced mainly by the changes in the intensity of bands characteristic of the stretching vibrations of the -OH group and the altered ratio of the 1720/1632 cm^−1^ bands, but also by the increased intensity of bands such as those with maxima at 1326, 1222, or 1019 cm^−1^. As follows from the literature data, mechanical strength usually cleaves only the amorphic region on the ordered surface in a crystalline substance [31,32,33]. Due to the same, the stiff and ordered structure of cellulose was slightly deteriorated by very fine grinding. All the mentioned shifts were related to the cleavage of hydrogen bonds present in the polymer chain during the grinding process. Additional cleavage of bonds and structural changes occurring in the polysaccharides were also facilitated by the observed increase in sample temperature during the micronization process. However, the observed spectral changes also clearly indicated that very fine grinding had no effect on the primary functional groups in cellulose, and the observed discrepancies were associated mainly with modifications to polysaccharide chains. This observation was further corroborated by the vibrations recorded below 930 cm^−1^, where the only effects noted related to band intensity. The bands in this region are characteristic of the vibrations on β-1,4-glycoside bonds, as already discussed above. As for the registered changes in the intensity of said vibrations, they evidence the same susceptibility to factors related to the micronization of the tested raspberry samples. 

As evidenced by the above, the use of FTIR spectroscopy allowed us to identify the bands of marker changes characterizing the micronization process employed. The presented results may allow for better optimization of the process in the course of future research with a view of obtaining even higher-quality products.

### 2.3. Identification of Sugars in Micronized Byproduct Raspberry Powders

**Figure 4:**
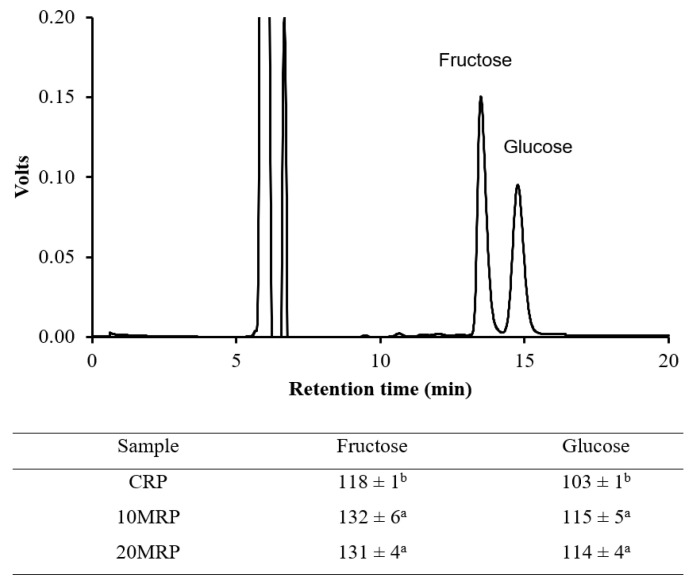
HPLC chromatogram and sugar content in micronized byproduct raspberry powders (mg/g). Values in the same column marked with different letters differ significantly (*p* < 0.05).

A study of the identification of sugars in raspberry powders (Figure 4) showed that, compared to control samples, micronization, regardless of the time of 10 or 20 min, resulted in significantly higher amounts of fructose and glucose. The disintegration of cell membranes during physical destruction, but also to some extent heat treatment, which facilitates the extraction of the solute, can be used to explain changes in the levels of sugars [42]. 

Additionally, our analysis of the FTIR spectra indicated that it is likely that the polysaccharides’ intramolecular hydrogen bonds had broken, boosting the proportion of simple saccharides in the mixture. According to a different study [43], micronizing soybean fibers resulted in a considerable reduction in the amount of polysaccharides, including cellulose. Research on the effects of ball mill micronization on the characteristics of polygonatum powder revealed that the process’ extension led to a much higher concentration of soluble sugar in these powders [44].

### 2.4. Identification of Phenolic Compounds in Micronized Byproduct Raspberry Powders

The list of compounds identified in this study using UHPLC-Q-TOFMS/MS is presented in Table 2, and the results of the content of individual phenolic compounds of micronized byproduct raspberry powders are shown in Table 3. 

**Figure 5:**
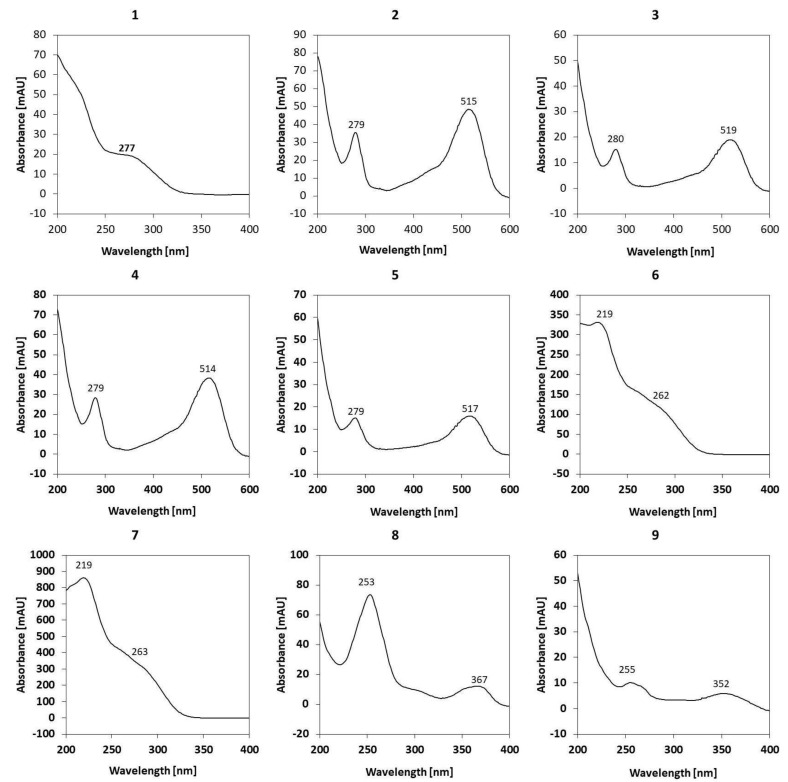
UV spectra of individual phenolic compounds of raspberry powders: **1**—ellagic acid derivative; **2**—cyanidin-3-sophoroside; **3**—cyanidin-3-(2-glucosylrutinoside); **4**—cyanidin-3-rutinoside; **5**—pelargonidin-3-rutinoside; **6**—ellagic acid derivative; **7**—ellagic acid derivative; **8**—ellagic acid; **9**—rutin.

The two main peaks showcased in Figure 5 can be attributed to ellagitannins such as lambertianin C, sanguiin H6, H10, casuarictin or their isomers. Similar results were obtained for red and black raspberry extracts [45,46]. Other researchers also confirmed that dimeric sanguiin H6 and trimeric lamberatianin C are the main ellagotannins in raspberries [47]. In the black raspberry seed extracts, lamberatianin C is not present [48]; however, in Siberian raspberries, they can be detected within the leave extracts [49]. 

Recorded UV spectrums for these compounds are depicted in Figure 5. We did not observe parent ions nor daughter ions above *m*/*z* 1235; however, according to the abovementioned team of Ross et al. [45], the peaks can be attributed to ellagotannins. It can be assumed that ion 934 is a daughter ion derived from the cleavage of complex ellagotannins [16].

The identification of phenolic compounds carried out showed that in the studied micronized powders from raspberry byproduct, there are nine different types of such compounds, including: ellagic acid derivative (**1**), cyanidin-3-sophoroside, cyanidin-3-(2-glucosylrutinoside), cyanidin-3-rutinoside, pelargonidin-3-rutinoside, ellagic acid derivative (**6**), ellagic acid derivative (**7**), ellagic acid and rutin. Significantly higher contents of ellagic acid and ellagic acid derivatives (**1**, **6**, **7**) and rutin were detected in micronized samples (10 and 20 MRP) than in the control (CRP) sample, regardless of micronization time in the tested range of 10 to 20 min. Longer micronization carried out for 20 min compared to the sample micronized for 10 min and the control sample reduced the content of cyanidin-3-rutinoside and pelargonidin-3-rutinoside. The content of the other phenolic compounds was not significantly different for all samples tested. In our previous studies [28] on wet and dry micronization of spinach leaves and stems, it was observed that both dry and wet micronization affected the contents of o-coumaric acid and gallic acid. In addition, dry micronization of spinach leaves increased the content of 3-hydroxyphenylacetic acid, 4-hydroxyphenylacetic acid, and p-coumaric acid. Other studies on micronization of grape pomace and fiber concentrates have explained that micronization increases the extractability of phenolic compounds, especially catechin and epicatechin [50].

**Figure 6:**
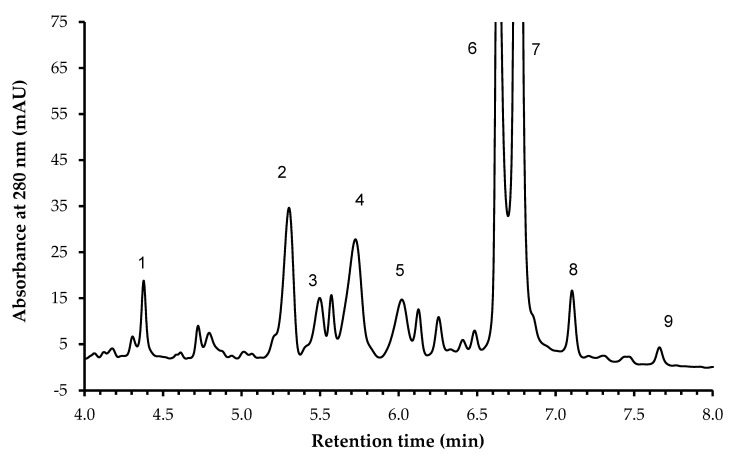
HPLC-DAD chromatogram of phenolic compounds of raspberry powder. **1**—ellagic acid derivative; **2**—cyanidin-3-sophoroside; **3**—cyanidin-3-(2-glucosylrutinoside); **4**—cyanidin-3-rutinoside; **5**—pelargonidin-3-rutinoside; **6**—ellagic acid derivative; **7**—ellagic acid derivative; **8**—ellagic acid; **9**—rutin.

We also found several anthocyanins that were tentatively identified by analyzing their fragmentation patterns. The presence of those compounds was also detected by RP-HPLC-DAD (Figure 6).

Other teams that have analyzed raspberries reported similar anthocyanin profiles [16,45]. The profile of the compounds that we observed in our study demonstrates that other compounds can be detected in raspberry pomace. We observed derivatives of phenolic acids and flavonoids, e.g., glucuronides, hexosides, and pentosides. The general profile of the compounds that are present in the raspberry pomace is comparable with other studies [16,45,46,49]. However, more flavonoid aglycones were recently identified in raspberry puree [51], probably due to the fact that most of them are removed when puree/juice is produced and when pomace is obtained.

### 2.5. Antioxidant Potential of Micronized Byproduct Raspberry Powders

The results of the total phenolic and antioxidant potential of micronized byproduct raspberry powders are shown in Table 4. 

After the micronization process, the antioxidant potential measured in our study by ABTS and FRAP greatly enhanced; there were no significant differences between the micronization times of 10 and 20 min. The scavenging activity index (ACL) did not significantly vary for any of the raspberry powder test samples. However, after the application of micronization, a considerable drop in the value of the DPPH indicator was seen. 

Whole raspberry micronized fruits obtained by fluidized bed jet milling with drying were characterized by a higher content of anthocyanins and polyphenols as well as by higher antioxidant properties compared to the powders obtained by convection and spray-drying methods [24]. 

Sheng et al. [31] found that grape pomace treated by superfine grinding treatment had lower total phenolic content and proanthocyanidins values than control samples. The heat degradation of phenolic compounds was indicated by the authors as the explanation for the decrease in the concentration of these compounds. In our investigation, micronizing raspberry powder for 10 min did not cause it to reach a temperature of 41 °C, and micronizing it for 20 min did not cause it to reach a temperature of 55 °C.

## 3. Materials and Methods

### 3.1. Materials

The raw material for the research was raspberries of the Polesie variety from a plantation in the Lublin region. Raspberry pomace as a byproduct during juice production was obtained with the use of a slow-running press. The research material was obtained by freeze-drying raspberry pomace. The resulting pomace was molded into dies and frozen at −30 °C under free convection conditions. In this way, frozen cuboidal solids with dimensions of 2 × 2 × 4 cm were obtained, which were subjected to a freeze-drying process at a pressure of 20 Pa for 72 h without heating the shelves in the Christ Alpha 2–4 LD plus device. The obtained lyophilisate was crushed and sieved to separate the seeds. In further analyses, only pomace without seeds was used.

### 3.2. Micronization of Raspberry Byproduct Powders

**Figure 7:**
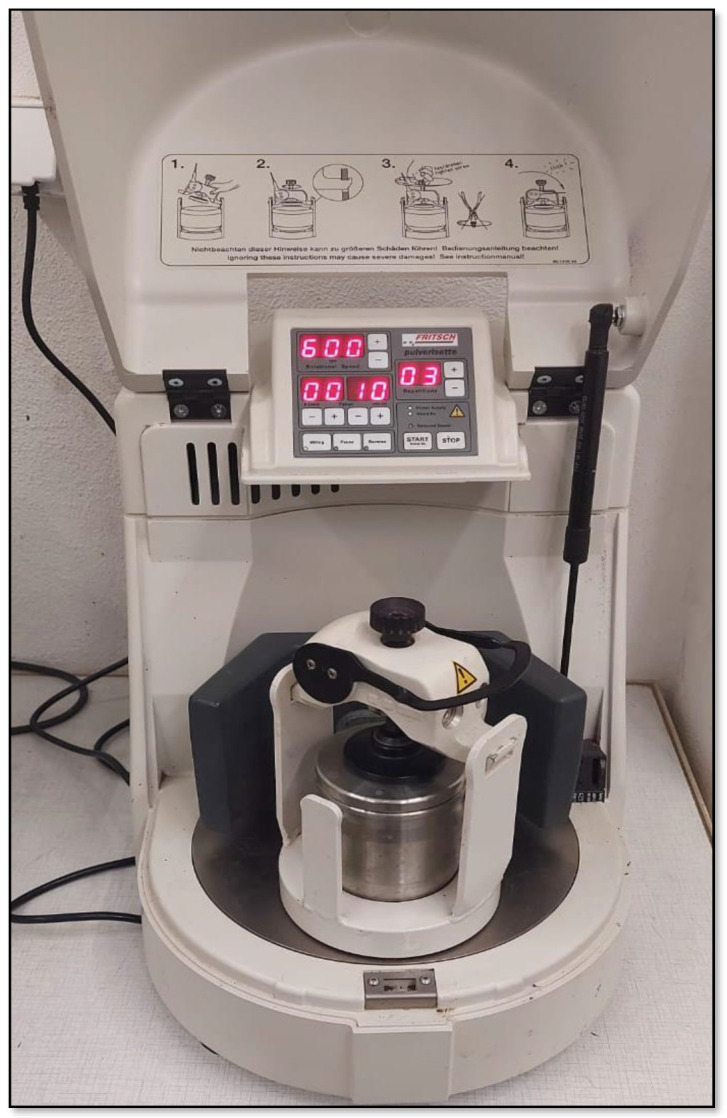
The appearance of the ball mill (Pulverisette 6) used for micronization.

The micronization of freeze-dried raspberry byproduct (without seeds) was carried out on a ball mill (Pulverisette 6, Fritsh, Idar-Oberstein, Germany) (Figure 7). The Planetary Mill with one working station uses one 500 ml grinding bowl that rotates with a transmission ratio of 1:1.82 relative to the main disk. The primary disk’s rotational speed can be adjusted from 100 to 650 revolutions per minute. Grinding balls with a diameter of 10 mm made of hardened, stainless steel, FE-CR, were utilized in the process. Freeze-dried raspberry pomace powder without seeds (50 g) was placed into a bowl filled with 15 balls and micronized for 10 or 20 min with a speed of 600 rpm. The process was carried out with simultaneous monitoring of the particle size and temperature after micronization. It was observed that 10 min of micronization already significantly reduced the particle size. Further extension of micronization to 10 min increased the temperature of the raw material to 41 °C, and after 20 min to 55 °C. We did not want to cause a large degradation of the compounds; thus, we finished the process after 20 min. As a result, three samples were chosen for further study: a control sample of freeze-dried non-micronized raspberry pomace (CRP), a sample of freeze-dried raspberry pomace micronized for 10 min (10 MRP), and a sample of freeze-dried raspberry pomace micronized for 20 min (20 MRP).

### 3.3. Particle Size Analysis

Analyses of the particle size of control raspberry pomace powder (CRP) and micronized raspberry powders (10 and 20 MRP) were performed on a Mastersizer 3000 (Malvern Instruments Ltd., Malvern, UK) [28,52]. Measurements were made using a dry dispersion adapter (Aero S). As a result of the measurements, the mean particle dimensions weighed by volume (D[4;3] (μm)) or surface area D[3;2] (μm) were obtained, and the dimensions of the specific surface area (SSA) (m^2^·kg^−1^) and the distributions were obtained. D50 is the particle size in microns at which 50% of the sample is smaller or larger. d10 is the particle size, with 10% of particles being smaller than this dimension. D90 is the particle size at which 90% of the particles in the sample are smaller than this value. Particle size analyses of raspberry powders were performed in three replications.

### 3.4. Color Measurements

The color measurements of the analyzed samples were determined on the CIE L*a*b*, scale using the Precise Color Reader (4 Wave CR30-16, Planeta, Tychy, Poland) colorimeter [48]. The L* parameter, meaning the brightness of the material, was in the range of 0–100. The a* color index ranged from −150 to +100, and negative values indicated green and positive values red. The b* index, determined in the range from −100 to +150, determined the share of blue color when it was negative and yellow when it was positive.

### 3.5. Infrared Spectra Measurements

Measurements of the ATR-FTIR spectra registered for the analyzed samples were performed using an IRSprit spectrometer from Shimatzu (Tokyo, Japan). An ATR (Attenuated Total Reflection) attachment in the form of a Zn Se crystal with adequate geometry (45°) was used to facilitate multiple internal reflections of the laser beam. The micronized powder samples were placed on the crystal. The spectrometer attachment allowed for a very exact measurement owing to the very precisely controlled contact between the sample and the crystal, with the possibility of regulating the amount of pressure. The attachment facilitates considerably more precise measurements in samples of this particular type. During the measurements, 24 scans were registered for each of the samples. Subsequently, the software automatically averaged the obtained spectra. Before and after each measurement, the crystal was spotless using ultrapure solvents. All the solvents were purchased from Sigma-Aldrich (Poznań, Poland). The spectra were registered within the range from 450 to 3800 cm^−1^ at a resolution of 2 cm^−1^. The spectral measurements were conducted at the Laboratory of the Department of Biophysics, Molecular Biophysics Institute, University of Life Sciences in Lublin. All the clearly discernible bands were associated with corresponding vibrations based on a detailed review of available literature as well as on information regarding the structure of the molecules present in the analyzed samples. All the spectra were processed and prepared for publication using Grams AI software (Version 9.1) from ThermoGalactic Industries (San Jose, CA, USA).

### 3.6. Sugar Identification

Sugars were extracted from raspberry seeds with hot 85% (*v*/*v*) methanol [53,54]. Individual sugars were determined using the HPLC method. Individual sugars were separated using an HPLC Shimadzu system (Shimadzu, Kyoto, Japan), which consisted of an SCL-10A controller, an LC-10AD pump, and a RID-10A detector. A portion of 20 μL of the extract was injected into a Luna Omega 3 μm SUGAR column (4.6 × 250 mm) (Phenomenex, Torrance, CA, USA). The flow rate of the mobile phase (acetonitrile–water, 25:75, *v*/*v*) was 1 mL/min. For calibration, the external standard method was used.

### 3.7. Phenolic Compounds Extraction

Phenolic compounds were extracted from raspberry byproduct powder, according to Tomas [51]. Raspberry powder (2 g) was extracted in 10 mL of methanol–water solution (75:25, *v*/*v*) containing 1% of formic acid in an ultrasonic bath for 15 min (Ultron U-509, Dywity, Poland). Then, the sample was centrifuged at 2700× *g* at 4 °C for 10 min, the supernatant was collected, and the sample was adjusted to 25 mL with the solvent used for extraction.

The content of total phenolic compounds in the extract was determined using Folin–Ciocalteou’s phenol reagent [55]. The results were expressed as gallic acid equivalents per gram of raspberry powder.

### 3.8. HPLC-DAD Analysis of Phenolic Compounds

Polyphenolic compounds were analyzed using RP-HPLC-DAD. Extracts were injected (1 µL) into the Shimadzu Nexera system that consisted of a degassing unit (DGV-20A 5R), two pumps (LC-30AD), an autosampler (SIL-30AC), column oven, PDA detector (SPD-M30A) and a controlling unit (CBM-20A). The flow rate was set to 1 mL/mL. Separation (Kinetex, SHIM-POL, Warsaw, Poland C18 2.6 µm, 100 A, 75 × 3 mm) was monitored at 280 and 520 nm and was conducted under binary gradient conditions. The eluents used were (A) water:acetonitrile:trifluoroacetic acid (95:5:0.1, *v*/*v*/*v*) and (B) acetonitrile:trifluoroacetic acid (100:0.1, *v*/*v*). The gradient was set up for eluent B as follows: 0–10 min: 0–18.8%; 10.5 min: 0%; 12 min: 0%. Peak areas were recorded and compared with those of ellagic acid and cyaniding-3-glucoside obtained from prepared calibration curves. Results were expressed as milligrams of the standard per gram of extract per gram of D.W.

### 3.9. Identification of Phenolic Compounds

To identify more of the compounds from raspberry pomace, samples were analyzed using an Exigent microLC 200 system coupled with a TripleTOF 5600+ mass spectrometer (AB Sciex, Framingham, MA, USA). Electrospray ionization was performed in positive and negative. The operating MS conditions were as follows: ion spray voltage: 4.5 kV; turbo spray temperature: 350 °C; nebulizer gas (GS1) and curtain gas flow rate: 30 L/min; heater gas (GS2) flow rate: 35 L/min; declustering potential (DP) and collision energy (CE) for the full-scan MS: 90 or −90 V and 10 or −10 eV, respectively; and for MS2 mode: 80 or −80 V and 30 or −30 eV, respectively. The TOF MS scan was scanned in the mass range of 100–1250 *m*/*z*. Compounds were separated using an Exigent Halo C18 column (0.5 × 50 mm, 2.7 µm; AB Sciex). The binary gradient that was employed consisted of 0.1% (*v*/*v*) formic acid in water (eluent A) and 0.1% (*v*/*v*) formic acid in acetonitrile (eluent B), and it was set up from 5 to 90% B within 3 min, maintained to 3.8 min and 5% within 4 min to finally be maintained to 5 min.

### 3.10. Antiradical Activity Evaluation

Antiradical activity against ABTS•+ and DPPH• was determined using the methods described by Re et al. [56] and Amarowicz et al. [57]. The results were expressed as millimoles of Trolox equivalents (TE) per gram of powder. The method of Benzie and Strain [58] was used for the determination of ferric-reducing antioxidant power (FRAP). The results were expressed as mmol Fe^2+^ per gram of raspberry powder.

### 3.11. Photochemiluminescence Assay

The scavenging activity of raspberry byproduct powder samples was evaluated by a photochemiluminescence (PCL-ACL) method [59] in which superoxide radical anions (O2•−) are generated from luminol. The reactions were carried out using kits from Analytic Jena, (Jena, Germany). Measurement was performed on a Photochem device with PCLsoft 5.1 software (Analytic Jena). The results were expressed as mmol of Trolox equivalents per g of raspberry powders.

### 3.12. Statistical Analysis

Measurements were made in triplicate, means and deviations were calculated, and other statistical analyses were performed in Statistica 12.0 (StatSoft, Kraków, Poland). One-way analysis of variance (ANOVA) was performed, and Tukey’s test was performed to determine the significance of differences (*p* < 0.05) between the means. Significantly different means were marked with different letters (a, b, c, etc.) in the figures and tables.

## 4. Conclusions

The conducted research proved that the micronization process of freeze-dried raspberry pomace byproducts with the applied ball mill caused significant changes in the particle size distribution of the tested samples. The use of micronization for 10 and 20 min allowed for a significant reduction of the d50 particle size from 225 μm (CRP) to 25 μm (10 MRP) and 10.5 μm (20 MRP).

The micronization procedure significantly increased the amount of recognized sugars, such as glucose and fructose, in the raspberry powders. Despite a large reduction in particle size, the amount of these two sugars increased by approximately 12 percent after 10 and 20 min of micronization. There were no significant differences in glucose and fructose content between these samples.

Significantly higher contents of ellagic acid and ellagic acid derivatives and rutin were detected in the samples micronized compared to the control sample. Longer micronization reduced the content of cyanidin-3-rutinoside and pelargonidin-3-rutinoside.

The antioxidant potential of ABTS and FRAP significantly increased after 10 min of micronization, and extending this process to 20 min did not cause significant changes. According to these investigations, controlling the raspberry byproduct micronization process can result in powders with higher antioxidant potential. As a result, these powders can be utilized to create innovative functional foods and dietary supplements. Because of their low humidity, such powders may be stored for an extended period and can be simply utilized in processing. Furthermore, because of their finely separated particles and intense color, they can be used as functional food colorants with high antioxidant activity. In turn, the most significant discrepancies in the registered FTIR spectra were observed in the bands with the maxima at ~1720, 1635, and 1326. The process of micronization of raspberry byproduct samples resulted in the cleavage of intramolecular hydrogen bonds in the polysaccharide molecules present in the samples.

The research findings have significant industrial implications because they can be used to create innovative micronized raspberry powder that can be used as nutritionally valuable dietary supplements. The reuse of this pomace as juice-processing byproducts is a significant environmental issue.

For future research, it will be beneficial to run broader analyses on a larger number of samples, such as different raspberry varieties, so that correlation relationships or PCA tests can be conducted. Furthermore, additional investigation of the physical properties of the powders during storage, as well as in vivo testing, should be carried out.

## Figures and Tables

**Table 1:** The location of the maxima of the FTIR absorption bands, with assignments of particular vibrations to the respective raspberry samples corresponding to the data in Figure 4, registered within the spectral range of 450–3700 cm^−1^.

FTIR	Type and Origin of Vibrations
Positioning of Band (cm^−1^)	
3312	ν(O-H) in H_2_O and *intra*-molecular hydrogen bonding
2914	ν(C-H) in CH_2_ and CH_3_ asymmetrical and symmetrical
2845	
1723	ν(C=O)
1635	ν(C=C) or/and δ (O-H) adsorbed H_2_O
1541	ν(C=C)
1407	δ(-OH in plane), δ(CH_2_), δ(C-H)
1363	
1326	δ(C-H) and δ (O-H)
1222	δ(C-H) and asymmetrical bridge oxygen stretching -OH in-plane bending
1146	asymmetrical in phase ring stretching and ν(C-O-C) and ν(C-O) and ring stretching modes
1017	
912/893/866	*β*-linkage of cellulose ring breathing and asymmetrical out of phase stretching -OH *out-of-plane* bending and CH_2_ rocking
813/772	
613/580/550/511	

Note: ν—stretching vibrations; δ—deformation vibrations

**Table 2:** Identification of the main phenolic compounds of raspberry powders by UHPLC-Q-TOFMS/MS.

Compound Number	Ionization	Compound Name	MS	MS/MS
**1**	[M − H]^−^	Ellagic acid derivative	571	301, 229
**2**	[M − H]^+^	Cyanidin-3-sophoroside	611	449, 287, 269
**3**	[M − H]^+^	Cyanidin-3-(2-glucosylrutinoside)	757	611, 287
**4**	[M − H]^+^	Cyanidin-3-rutinoside	449	287
**5**	[M − H]^+^	Pelargonidin-3-rutinoside	579	271
**6**	[M − H]^−^	Ellagic acid derivative	934	1235, 934, 633, 315, 301
**7**	[M − H]^−^	Ellagic acid derivative	934	1235, 934, 633, 315, 301
**8**	[M − H]^−^	Ellagic acid	301	229
**9**	[M − H]^−^	Rutin	609	301

**Table 3:** Content of individual phenolic compounds, total phenolics and antioxidant potential of micronized byproduct raspberry powders (mg/g).

Compound Number	Compound Name	CRP	10 MRP	20 MRP
**1**	Ellagic acid derivative	0.132 ± 0.003 ^b^	0.161 ± 0.010 ^a^	0.166 ± 0.008 ^a^
**2**	Cyanidin-3-sophoroside	0.354± 0.001 ^a^	0.372 ± 0.014 ^a^	0.347 ± 0.013 ^a^
**3**	Cyanidin-3-(2-glucosylrutinoside)	0.134 ± 0.001 ^a^	0.142 ± 0.005 ^a^	0.132 ± 0.005 ^a^
**4**	Cyanidin-3-rutinoside	0.402 ± 0.003 ^a^	0.418 ± 0.015 ^a^	0.385 ± 0.010 ^b^
**5**	Pelargonidin-3-rutinoside	0.162 ± 0.001 ^a^	0.169 ± 0.006 ^a^	0.157 ± 0.002 ^b^
**6**	Ellagic acid derivative	0.697 ± 0.024 ^b^	1.034 ± 0.068 ^a^	0.878 ± 0.082 ^a^
**7**	Ellagic acid derivative	2.164 ± 0.041 ^b^	2.631 ± 0.120 ^a^	2.627 ± 0.128 ^a^
**8**	Ellagic acid	0.079 ± 0.001 ^b^	0.093 ± 0.004 ^a^	0.098 ± 0.003 ^a^
**9**	Rutin	0.012 ± 0.001 ^b^	0.013 ± 0.001 ^a^	0.014 ± 0.001 ^a^

Values in the same row marked with different letters differ significantly (*p* < 0.05). Content of compounds **1**, **6**, and **7** is expressed as ellagic acid equivalents, and compounds **2**, **3**, **4**, and **5** as cyaniding-3-glucoside.

**Table 4:** Total phenolics and antioxidant potential of micronized byproduct raspberry powders (mg/g).

Antioxidant Potential	CRP	10 MRP	20 MRP
Total phenolics (mg GAE/g)	19.74 ± 0.055 ^b^	22.79 ± 0.78 ^a^	23.94 ± 0.95 ^a^
ABTS (mmol TE/g)	0.180 ± 0.005 ^b^	0.221 ± 0.004 ^a^	0.216 ± 0.005 ^a^
DPPH (mmol TE/g)	0.241 ± 0.004 ^a^	0.201 ± 0.004 ^c^	0.218 ± 0.007 ^b^
FRAP (mmol Fe^2+^/g)	0.151 ± 0.003 ^b^	0.181 ± 0.004 ^a^	0.179 ± 0.004 ^a^
ACL (mmol TE/g)	0.183 ± 0.006 ^a^	0.178 ± 0.003 ^a^	0.180 ± 0.007 ^a^

Values in the same row marked with different letters differ significantly (*p* < 0.05).

## Data Availability

Not applicable.

## Sample Availability

Not available.
